# Mining for coexpression across hundreds of datasets using novel rank aggregation and visualization methods

**DOI:** 10.1186/gb-2009-10-12-r139

**Published:** 2009-12-04

**Authors:** Priit Adler, Raivo Kolde, Meelis Kull, Aleksandr Tkachenko, Hedi Peterson, Jüri Reimand, Jaak Vilo

**Affiliations:** 1 Institute of Molecular and Cell Biology, Riia 23, 51010 Tartu, Estonia; 2 Institute of Computer Science, University of Tartu, Liivi 2-314, 50409 Tartu, Estonia; 3 Quretec, Ülikooli 6a, 51003 Tartu, Estonia

## Abstract

We present a web resource MEM (Multi-Experiment Matrix) for gene expression similarity searches across many datasets. MEM features large collections of microarray datasets and utilizes rank aggregation to merge information from different datasets into a single global ordering with simultaneous statistical significance estimation. Unique features of MEM include automatic detection, characterization and visualization of datasets that includes the strongest coexpression patterns. MEM is freely available at http://biit.cs.ut.ee/mem/.

## Abstract 2

The MEM web resource allows users to search for co-expressed genes across all microarray datasets in the ArrayExpress database.

## Rationale

During the last decade, the gene expression microarrays have become a standard tool in studying a large variety of biological questions [1]. Beginning from the first experiments [2], microarrays have been used for pinpointing disease-specific genes and drug targets [3,4], uncovering signaling networks [5], describing cellular processes [6], among many other applications. While the methods for single experiment analysis are well established and popular [7], it is clear that information extracted from a single experiment is constrained by details of experimental design such as conditions and cell types. Integrating data from different experiments widens the spectrum of biological conditions and increases the power to find subtler effects.

Coexpression is one of the central ideas in gene expression analysis. The 'Guilt by association' principle states that gene coexpression might indicate shared regulatory mechanisms and roles in related biological processes. The validity of the principle is proved in several studies, see for example [8-10]. The idea can be applied in many tasks of computational biology, such as inferring functions to poorly characterized genes [9,11,12], discovering new putative members for metabolic pathways [12], or predicting and validating of protein-protein interactions [13,14]. Many *de novo *regulatory motif discovery methods use gene expression similarity information as a primary input for identifying co-regulated genes [15,16]. More recently, gene expression similarity search has been utilized in a pathway reconstruction study [17].

Multi-experiment coexpression analysis can be a labour-intensive and computationally challenging task. First steps involve collecting suitable datasets, data downloads, preprocessing, normalization, and gene annotation management. Then, methodological and technical questions arise, namely the integration of different datasets, merging cross-platform data, and handling ambiguous mappings between genes and probesets. Finally, the sheer size of targeted data requires efficient computational strategies or caching of pre-calculated results. The complexity of multi-experiment microarray analysis is likely its main limitation, as researchers often lack the time and resource to take on such a task. Consequently, there is a clear need for services that provide coexpression information in an easy and accessible format.

Surprisingly, the resources and tools for finding genes with similar expression profiles in multiple experiments are still rather scarce.

Microarray databases ArrayExpress [18] and Gene Expression Omnibus (GEO) [19] have implemented a data mining layer for finding and analyzing most relevant datasets, but neither yet provides a comprehensive gene coexpression search over many datasets simultaneously. Gemma is a web based resource that utilizes a global inference strategy to detect genes that have similar expression profiles in all covered datasets [20]. However, global coexpression analysis is likely to miss similarities that occur in a tissue or condition specific manner [21]. SPELL is a resource that puts a strong emphasis on selecting the appropriate datasets for the query [22]. The method identifies the subset of most relevant datasets by analyzing the coexpression of a user-defined list of genes, and uses the subset to find additional genes. Unfortunately, detecting relevant datasets relies on the user's knowledge of genes that are likely to have similar expression profiles. Furthermore, it currently features relatively small number of datasets, all of them describing yeast.

We have developed the query engine MEM that detects coexpressed genes in large platform-specific microarray collections. The Affymetrix microarray data originates from ArrayExpress and also includes datasets submitted to GEO and automatically uploaded to ArrayExpress. MEM encompasses a variety of conditions, tissues and disease states and incorporates nearly a thousand datasets for both human and mouse, as well as hundreds of datasets for other model organisms.

MEM coexpression search requires two types of input: first, the user types in a gene ID of interest, and second, chooses a collection of relevant datasets. The user may pick the datasets manually by browsing their annotations, or allow MEM to make an automatic selection based on statistical criteria such as gene variability. MEM performs the coexpression analysis individually for each dataset and assembles the final list of similar genes using a novel statistical rank aggregation algorithm. Efficient programming guarantees rapid performance of the computationally intensive real-time analysis that does not rely on precomputed or indexed data. The results are presented in highly interactive graphical format with strong emphasis on further data mining. Query results and datasets can be ordered by significance or clustered. The MEM visualization method helps highlights datasets with highest coexpression to input gene and helps the user distinguish evidence with poor or negative correlation. Datasets are additionally characterized with automatic text analysis of experiment descriptions, and represented as word clouds that highlight predominant terms. With MEM we aim to make multi-experiment coexpression analysis accessible to a wider community of researchers.

## MEM web interface

### Input

#### Primary input

The primary input of MEM is a single query gene that acts as the template pattern for the coexpression search. The tool recognizes common gene identifiers and automatically retrieves corresponding probesets, the conversion is based on g: Profiler [23] and Ensembl [24] ID mappings. When several probesets link to a gene, the user needs to choose one of the probesets for further analysis.

Second, the user needs to select the collection of datasets where similarities between expression profiles are detected (the search space). ArrayExpress datasets are organized into platform-specific collections and the user may choose perform the search over all datasets of a specific platform. The search space may be further narrowed by browsing dataset annotations and composing a collection that covers a specific disease or tissue type.

#### Dataset selection

In multi-experiment coexpression analysis, some individual datasets may produce noisy or even entirely random results that are either caused by poor data quality or low expression levels of the query gene. The quality of the analysis can be improved considerably by eliminating the datasets that create a noise bias for the query gene. Low dataset-wide variability of expression levels is one of the key indicators of spurious results. Minute changes in gene expression are often caused by experimental noise rather than cellular mechanics. Therefore, corresponding similarity searches are likely to be less informative about gene function.

We have included a standard deviation filter in the MEM interface that allows the users to detect and disregard datasets where the variability of the query gene is low. Based on extensive simulations detailed in the Methods section, we conclude that the standard deviation *σ *= 0.29 is a reasonable threshold for distinguishing informative datasets. The above filter holds for the entire analysis since all related datasets are normalized and preprocessed using the same algorithm.

#### Search algorithm parameters

The first step of MEM multi-experiment coexpression analysis detects the most similar candidate genes for each individual dataset. The most important parameter for this stage is the distance measure that defines the similarity between expression profiles and has a significant impact on the contents and interpretation of results. Pearson correlation is the default distance measure in MEM. It evaluates the dynamic similarity of expression profiles and has become a standard method of measuring coexpression [25]. Another useful measure is the anti-correlation distance that detects inverse expression patterns, such as genes responding to repressor activity. For example, anti-correlation queries have been used to validate predicted micro RNA targets [26]. Absolute correlation distance is a combination of the above measures, as it detects both direct and inverse similarity.

**Figure 1:**
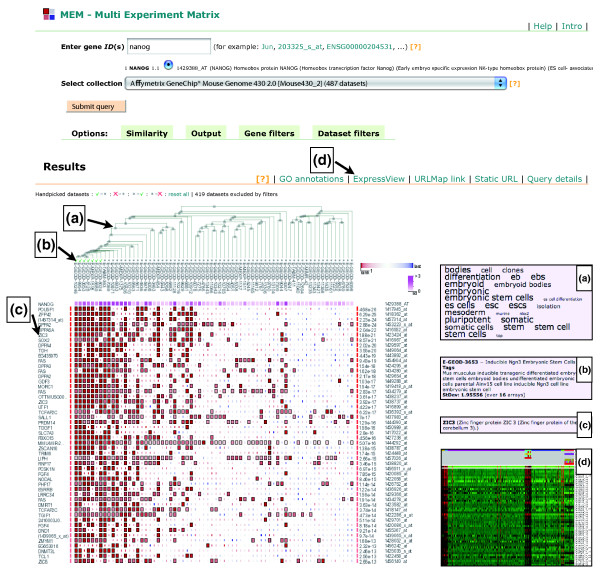
MEM user interface and results for the transcription factor *NANOG*. The top of the page contains controls for the query: gene input field, dataset selection and advanced options. Bottom of the page shows the results of the query. The genes, which are displayed as rows, are ordered by multi-experiment similarity to the query gene. Additionally, the single experiment similarity ranks are displayed as a matrix of colored squares, where red and blue denote small and large ranks, respectively. The larger squares indicate the ranks that contributed to the final *P*-value. Each element corresponds to a experiment and the columns are clustered. Hovering over the results brings up context specific information: **(a) **word cloud that characterizes the corresponding experiments; **(b) **single dataset annotations; **(c) **gene names with short descriptions. The row of links above the results facilitates the further analysis of results. For example, the user can visualize the expression of selected datasets (marked with green ticks) as a heat map **(d)**.

After detecting the most similar genes in individual datasets, we apply a novel rank aggregation algorithm that merges candidates of different datasets and creates the final list of coexpressed genes. The rank aggregation algorithm assigns a *P*-value to each gene, in order to evaluate its similarity to the query gene across the given collection of datasets. Statistically, the *P*-value reflects the likelihood of the gene appearing with certain observed ranks in the datasets if the similarity lists were shuffled randomly. Selecting the expression profiles with most significant *P*-values accurately retrieves genes with high expression similarity and functional relevance to the query gene (Figure 1).

### Output

The principal output of MEM is a ranked list of genes that are coexpressed with the query gene in the provided datasets. For each resulting gene, MEM provides a *P*-value that reflects the significance of its expression similarity to the query gene across the collection on analyzed datasets. A wealth of interesting information is presented in the graphical rank matrix (Figure 1). Each column of the matrix stands for a dataset, each row represents a gene, and each matrix element reflects the individual similarity rank for the given gene in the given dataset. Visual inspection of the rank matrix allows the researcher to detect patterns of correlation across datasets and spot significantly stronger coexpression profiles. The rank aggregation algorithm provides a natural cutoff between informative and non-informative ranks for each gene. Colors and cell size is used to highlight datasets where the given gene was particularly similar to the query gene and hence contributed significantly to the final *P*-value.

Genes with the greatest similarity rankings are frequently in strong correlation only within a relatively small fraction of datasets that are biologically relevant to gene function. If the contributing datasets can be related in the context of experimental design, one may learn additional information about the query gene and its association to the resulting genes. Columns of the rank matrix are clustered hierarchically, so that datasets with similar correlation patterns are grouped together using a tree visualization, and datasets with most impact are aligned to the left. While the default policy is to filter datasets based on the standard deviation criterion, one may take advantage of the high contribution of few datasets and manually remove experiments that have little impact on the final list of correlated genes. Single clicks on datasets or tree nodes toggle whether selected experiments or entire experiment groups are regarded in downstream analysis.

A text mining technique called word cloud gives a compact semantic overview of a selected group of datasets through the descriptions of experimental designs. The word cloud detects keywords that are enriched in the experimental descriptions of the group, and uses different font sizes to highlight terms with strong statistical significance. One may study the experiment descriptions of single datasets and dataset clusters by moving the mouse over the dataset clustering tree.

Additional features of the tool reveal finer details of underlying data and create multiple pointers for further analysis. Besides coexpression associations in the rank matrix, MEM also displays standard heat maps with expression profiles and experimental details of individual datasets. The heat maps provide an easy visual validation of detected coexpression patterns. MEM includes filters that constrain the output to certain genes and allow the researcher to seek answers to interesting problems. For instance, one may study the association of the query gene in relation to a certain pathway or biological process, by comparing the expression patterns of its members. The URLMap feature provides easy access to external resources, as it automatically links resulting genes to multiple genomic databases [27]. Coexpressed genes can be directed to the g: Profiler toolset for functional enrichment analysis of Gene Ontology terms, pathways and *cis*-regulatory motifs [23].

## Case studies

### MEM query with embryonic stem cell regulator NANOG retrieves ES cells related genes and datasets

The homeobox transcription factor *NANOG *is a key regulator of differentiation and pluripotency maintenance in mammalian embryonic stem cells [28,29]. *NANOG *forms a complex circuitry together with the factors *OCT4 *and *SOX2 *and is involved in the combinatorial regulation of a range of downstream developmental processes.

We demonstrate the power of the MEM toolset by analyzing the genes that show strong coexpression patterns with *NANOG *across multiple datasets (see Figure 1). We chose a collection of 487 mouse datasets of the Affymetrix 430-2 platform, as the platform includes the largest amount of ES cells related experiments. After applying the default standard deviation filter (*σ *= 0.29), MEM automatically removed 419 datasets where the expression level of *NANOG *was insufficient for coexpression analysis. As the role of *NANOG *role is believed to be restricted to embryonic stem cells only, datasets covering other tissues and conditions are expectedly uninformative and provide no results of statistical significance (data not shown). On the other hand, datasets considered relevant by MEM appear to be related to the role of *NANOG*. Keyword analysis of experimental annotations reveals enriched terms like 'embryonic', 'pluripotent', 'stem cell' and so on (see word cloud, Figure 1a).

In response to the *NANOG *query, MEM retrieves a list of coexpressed genes that appear to be functionally related to embryonic stem cells. Enrichment analysis with top 50 probesets reveals important functional terms from Gene Ontology (for example, stem cell development *P *< 10^-12 ^and regulation of transcription *P *< 10^-6^). The top list includes key transcription factors *OCT4 *(position 1) and *SOX2 *(position 7) as well as other genes with known roles in stem cell regulation and maintenance of pluripotency. For instance, UTF1 is a ES cell specific transcriptional coactivator [30], while *DPPA2/3/4/5A *are nuclear factors with a role in regulating pluripotency [31]. *NODAL *is a member of the TGF-beta superfamily whose signaling is required for maintaining pluripotency in human embryonic stem cells [32]. Signaling of *TDGF *(Cripto) in a *NODAL*-dependent manner directs the differentiation and fate determination of ES cells [33]. *TGF3 *is another growth factor that has been shown to involve in the patterning of the anterior-posterior axis and exhibit signaling similar to NODAL [34].

**Figure 2:**
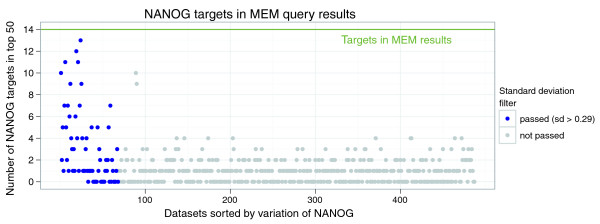
*NANOG *targets among first 50 MEM results. MEM query with transcription factor *NANOG *retrieves more of its targets among top 50 genes, than queries on any one dataset individually. Each point represents the overlap between *NANOG *targets and top 50 query results in one of the 487 datasets. The datasets are sorted by variation and the ones that pass standard deviation filter are highlighted. Most of the datasets that retrieve high number of *NANOG *targets pass the filter, which shows the specificity of the filter.

In a previous study, Sharov *et al*. inferred direct targets of *NANOG *by computational integration of gene expression and chromatin immunoprecipitation data [35]. 14 of the 281 targets of the above study are also detected by MEM among top-50 most significant genes (*P *< 10^-13^). To put this result into context, we performed a similarity search in each of the 487 datasets individually, and found that each dataset yielded a smaller number of targets than the composite MEM query (Figure 2). To show the utility of the standard deviation based filter, we highlighted the datasets that passed the filter. Only 20 out of 487 datasets had overlap larger then 4 and only two of them did not pass the standard deviation filter, confirming the accuracy of the filter in selecting relevant datasets.

### Analysis of MEM coexpression network reveals functional modules of cell cycle, proteasome and the immune system

Coexpression information can be used to reconstruct biological networks and regulatory pathways [36-38]. In such a network, genes act as network nodes, that are associated via edges if their expression patterns are in strong correlation. Coexpression networks have been shown to contain densely connected modules that include genes of related function [10].

We used MEM to build a coexpression network of the mouse genome, using a collection of 89 datasets (Additional file 1) of the Affymetrix U74Av2 platform as the search space. In the first stage, we retrieved the list of coexpressed genes for every mouse gene, and constructed the network by connecting gene pairs where both genes of the pair had significant MEM similarity scores with one another. After applying a Bonferroni multiple testing correction, we ended up with a dense network with 115664 edges between 5440 genes with statistical significance below 0.001. In the second stage, we applied the Markov Cluster (MCL) algorithm [39] via the GraphWeb tool [40] to prune the network and find gene modules. The MCL algorithm simulates a stochastic flow in the expression graph and removes edges that are visited infrequently, resulting in a collection of densely connected groups of genes. In the third stage, we assessed the functional relevance of detected modules with GraphWeb, by finding significantly enriched Gene Ontology terms (GO), Kyoto Encyclopedia of Genes and Genomes (KEGG) and Reactome biological pathways, and cis-regulatory motifs.

**Figure 3:**
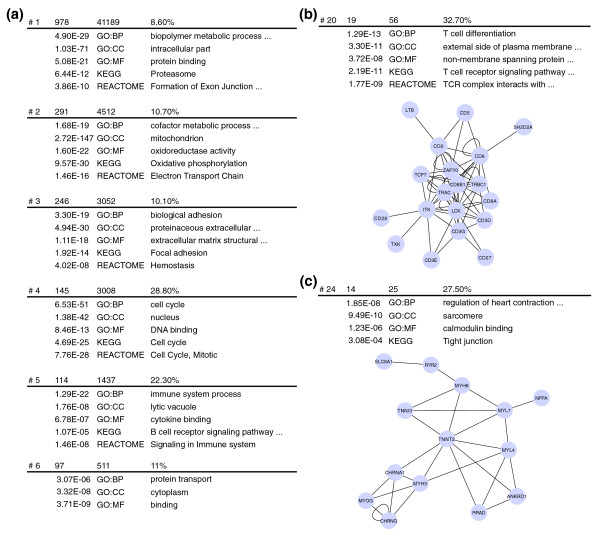
Functional descriptions of the modules found in the mouse coexpression network constructed with MEM. Annotations of the six largest modules are shown in **(a)**. Two smaller modules are shown in the Figure, along with their functional annotations in **(b) **and **(c)**.

The size, density and functional descriptions of the six largest modules can be seen on Figure 3a. All have strong and clear functional annotations, that is, proteasome (KEGG, *P *< 10^-11^), mitochondria (GO, *P *< 10^-146^), cell cycle (GO, *P *< 10^-50^), biological adhesion (GO, *P *< 10^-18^), immune system process (GO, *P *< 10^-21^) and protein transport (GO, *P *< 10^-5^). Several smaller modules with interesting functional annotations are also detected, for instance one related to T-cell generation (Figure 3b, *P *< 10^-12^) and one related to regulation of heart contraction (Figure 3c, *P *< 10^-7^).

### MCM complex of DNA replication initiation shows consistent expression patterns with ORC, GMNN and CDC6L/45L

Stable protein complexes are made up of several physically interacting proteins. In order to keep essential complexes intact, corresponding subunits need to have consistent expression patterns across many diverse conditions and tissues. Hence, a MEM query with a selected complex subunit should retrieve the remaining complex subunits with high ranks. Queries with different subunits are expected to retrieve similar lists of well-correlated genes whose functional role is related to that of the complex in question. In order to validate MEM performance on protein complexes, we studied the expression patterns of the essential *MCM *(Mini Chromosome Maintenance) complex that is conserved in eukaryotes from yeast to human. *MCM *is involved in the regulation of DNA replication during cell cycle, a complex multistep process that involves the cooperation of a number of proteins [41]. *MCM *is a helicase of six subunits (*MCM2*-*MCM7*) that forms the Pre-Replicative Complex (preRC) together with the Origin Recognition Complex (*ORC1*-*ORC6*) and cell division cycle proteins (*CDC6*, *CDC45*) [42]. The preRC binds to the origins of recognition on the DNA and initiates replication during the G1 phase of the cell cycle. The *MCM *complex acts as the licensing factor of replication, ensuring that DNA is synthesized only once per cell cycle [43]. Besides initializing DNA replication, *MCM *also has a later role during DNA synthesis in strand elongation. The presence of the complex appears to be correlated with cell proliferation and suggests roles in cancer [44-46].

We composed a compendium of 145 cancer-related microarray datasets (Additional file 2) of the human Affymetrix U133A platform from ArrayExpress to analyze the expression profiles of *MCM *complex subunits *MCM2*-*MCM7*. For each of the *MCM *subunits, we used MEM to retrieve a ranked list of 100 probesets with most correlation relative to the subunit, referred to its cohort. In case of multiple probesets corresponding to a subunit, we picked the probeset whose cohort contained most cell cycle related genes. We excluded *MCM7*, as the corresponding probeset also maps to several unrelated genes.

The subunits of the *MCM *complex have extremely consistent expression profiles across the compendium of cancer-related datasets. Among the cohorts of *MCM *subunits, other *MCM *probesets are always delivered with a high rank (median rank 17.5). The *MCM *cohorts are generally very similar, as on average, a pair of *MCM *subunits shares 65 probesets of the 100-element cohorts and the six 100-probeset cohorts contain a total of 116 probesets that occur in more than two cohorts (Additional file 3). These overlaps are very unlikely to occur by random chance, as even the protein pair with least common probesets has a highly significant *P*-value (*MCM5 *and *MCM6*, 47 common probesets, *P *< 10^-87^).

MEM coexpression patterns are functionally well reflected in the cohorts. The probesets have strong enrichments that are related to the role of the *MCM *complex as well as the cancer-specific context of the analyzed datasets. g: Profiler reveals enrichments of generic terms such as the cell cycle (GO, *P *< 10^-42^) and DNA replication (GO, *P *< 10^-37^), as well as more specific functions like DNA replication pre-initiation (Reactome, *P *< 10^-11^) and DNA strand elongation (Reactome, *P *< 10^-21^). The promoters of coexpressed genes have enrichments for the binding site of *E2F1*, a transcription factor with a recognized role in replication regulation and oncogenesis (for example, Transfac, M00427, consensus sequence TTTSGCGS, *P *< 10^-6^) [47,48]. The enrichment in the *P53 *pathway (KEGG, *P *= 10^-4^) suggests a link with the well-identified tumor suppressor gene [49]. Moreover, the cohorts contain microRNAs as well as enrichments for microRNA target sites that may have cancer-specific roles. For instance, the coexpressed genes have a greater than expected proportion of target sites for the microRNA *miR-142-5p *(miRBase, *P *< 10^-4^), a regulatory RNA that has been detected in the context of leukemia [50].

**Figure 4:**
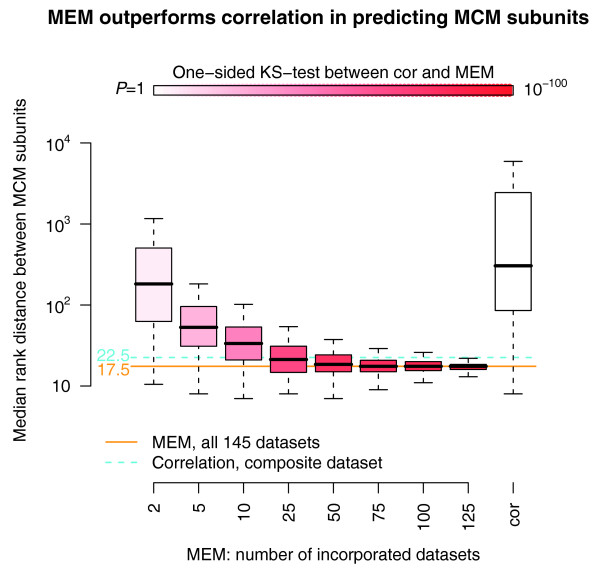
Increasing the number of datasets for MEM queries improves prediction of Mini Chromosome Maintenance (*MCM*) subunits. As additional datasets are incorporated for MEM analysis, *MCM *complex subunits show more consistent expression patterns as measured by median distance between subunits in MEM ranked lists of most correlated genes (decreasing bar height). According to one-sided Kolmogorov-Smirnov tests, MEM analysis with different numbers of datasets (left bars) significantly outperforms correlation (rightmost bar). In addition, MEM analysis for all the 145 selected datasets gives improved results compared to plain correlation across the concatenated dataset (light blue and orange lines).

In order to investigate the advantage of MEM analysis for coexpression over multiple datasets, we conducted a computational experiment where varying numbers of datasets were incorporated for delivering *MCM *cohorts (Figure 4). For each of the sample sizes ranging from 2 to 125, we used 300 randomized collections of input datasets from the above cancer compendium to measure the median distance between *MCM *subunits in individual cohorts. As expected, adding more datasets into MEM analysis brings *MCM *subunits closer in resulting ranked gene lists. According to the Kolmogorov-Smirnov one-sided test, using MEM queries over several datasets always gives significantly better results (for example, increased similarity between *MCM *subunits) than correlation over any of the datasets individually. The advantage of MEM analysis appears to increase exponentially in relation to analyzed datasets. Importantly, the MEM query over all 145 cancer-specific datasets provides a smaller median distance between *MCM *subunits (*m *= 17.5), compared to the correlation over the concatenation of corresponding datasets (*m *= 22.5).

## Conclusions

As the amount of publicly available microarray data grows, methods that extract useful information from multiple datasets become ever more valuable. However, without specialized tools, the task of analyzing hundreds of datasets can be very labour-intensive. With the development of the MEM resource we have solved many of the technical challenges and aim to make high-throughput coexpression mining accessible for a larger audience.

MEM includes a large collection of up-to-date microarray datasets from the ArrayExpress database. We have developed a flexible strategy for coexpression analysis that puts great emphasis on selecting the most appropriate datasets for the query and uses a novel statistical algorithm to detect significant correlation patterns. Finally, MEM results are presented in an interactive graphical user interface that opens up several paths for further data analysis.

Still the MEM analysis has some limitations and possibilities for further development. The main limitation of the tool is the lack of across-platform similarity search, that is due to the complexity of mappings between probesets of different platforms, and comparability of normalizations. Fortunately, the number of various platforms for each model organism is relatively low and the bulk of experiments is often available in a single platform. In a number of network reconstruction applications, one might be interested in the coexpression of units of multiple genes such as protein complexes. Therefore, providing methods that allow comparison of groups of genes would be a natural development of MEM.

## Methods

### Rank aggregation

Rank aggregation is the heart of MEM coexpression analysis. It uses the statistical distribution of orderings to integrate individual lists of similar genes into final lists with significance *P*-values for each gene. The rank aggregation problem has been studied mainly in the context of voting and social choice, but there are also several bioinformatics applications, for example, [51,52].

Most classical methods assume that each individual ranking is reasonable and should be taken into account in composing the final ordering. However, in the case of gene coexpression analysis, some rankings include considerable amounts of noise as they are derived from genes and conditions with low variation. In order to overcome this, we first identify reliable gene lists that are based on sufficient variation, and then compute the rank aggregation based on the limited set of lists.

The input of rank aggregation is a collection of ordered lists, where every element in a list corresponds to a gene in a specific experiment, showing the rank of similarity to the query gene g*, relative to all other genes in the organism. We normalize the lists into the range [0.1], by dividing each individual rank by the maximal rank, that is, the number of genes in the microarray platform. We transform the ranks so that for each gene *g*_*i*_, we have a rank vector *r*(g*, g_*i*_) = [, ..., ] where  corresponds to the position of *g*_*i *_in the query on dataset *j*.

A straightforward solution for rank aggregation involves reordering the genes *g*_*i *_based on their arithmetic means of individual ranks *r*(g*, g_*i*_). Unfortunately this approach is rather sensitive to noise, since the mean is heavily influenced by large ranks that indicate no strong correlation. Geometric mean is more sensitive to small ranks and robust to fluctuations among large uninformative ranks. An alternative and empirically more successful approach uses trimmed mean that only considers *k *smallest elements, but requires the estimation of the parameter *k*.

We developed a statistical strategy for robust rank aggregation that overcomes the problems of mean-based methods and allows us to evaluate the statistical significance of detected similarity. As a null hypothesis, we consider a model ranking where similar genes are permuted randomly and the distribution of each rank vector *r*(g*, g_*i*_) is approximately uniform. In the biological case of strong coexpression, we observe an unexpectedly large amount of small ranks between genes with correlated expression patterns, so that the distribution of *r*(g*, g_*i*_) is skewed towards small values and significantly different from a uniform distribution. We can reorder the rank vector *r*(g*, g_*i*_) increasingly to gain the vector of order statistics  which range from the smallest to the largest value of *r*(g*, *g*_*i*_). Assuming the null hypothesis, we can use the binomial distribution to calculate the probability that *k *or more ranks are smaller than , for every *k*:(1)

The final similarity score *ρ *between g* and *g*_*i *_is defined as follows:(2)

In other words, for every value of *k*, we compute the *P*-value for each rank statistic *r*_(*k*) _being randomly as small as observed in the dataset, and as a final score we use the minimal *P*-value.

The final *ρ *score itself is not a *P*-value, since it is a minimum of *P*-values. Still, we may use a multiple testing correction to remove false positives that occur due to several independent tests. As we calculate the *ρ *scores for each gene, we actually find a *P*-value corresponding to each rank matrix element. According to Bonferroni correction for multiple testing, an individual *P*-value is significant if it is smaller than the desired significance level after multiplication by the number of rows and columns of the rank matrix. We cannot use any less stringent criteria for correction, since *P*-values for the same gene are strongly correlated.

As a byproduct of the above computation, we gain information about the datasets that contain significant coexpression between any two genes. A dataset with a ranking  that is smaller than the ranking that gave rise to *ρ*(g*, *g*_*i*_) can be considered significant. This feature allows us to highlight the contributions of different datasets into the final similarity ranking, and observe interesting patterns between related datasets. The score *ρ *also has the advantage of being non-parametric, as it makes no requirements on the number of input datasets or the magnitude of relevant ranks. In a way our *ρ*-score represents a natural balance between two scenarios: a gene that strongly correlates with the query gene in a small number of samples, and a gene that shows weak correlation in a large range of samples.

### Microarray data

All data used in the analyses has been obtained from ArrayExpress and it also includes datasets that were originally submitted to GEO. We only included Affymetrix datasets where raw data was available, and performed a uniform Robust Multi-array Average (RMA) normalization [53] with the Bioconductor *affy *package [54] using the default parameters. MEM also includes biological annotations of the datasets as annotated according to the Minimum Information About a Microarray Experiment (MIAME) standard [1]. The annotations are used for building word clouds and annotation tracks in heat map visualization of gene expression data.

### Standard deviation threshold selection

We performed a simulation study to find the threshold for query gene variation that would best identify the datasets where the gene has meaningful expression patterns. All the experiments in MEM are normalized and preprocessed the same way, so we may compute a uniform threshold that applies to all datasets. In the simulation, we chose random sets of 2000 genes and 140 experiments on human Affymetrix platform HG-U133A, and calculated the standard deviation for each gene in each experiment. We also performed a MEM query with each of the genes and used similarity score cutoff that yielded on average 20 genes per query. Now we tried several thresholds for the standard deviation and in each case we calculated correlation between the number of experiments exceeding the threshold and the number of genes in the result of the query. We achieved strongest coexpression patterns between the query genes and the resulting genes when using a standard deviation cutoff between 0.25 and 0.39, while the peak performance was observed at the threshold 0.29 (Additional file 4).

### Dataset annotation word cloud

MEM uses word clouds to display aggregated annotations of multiple datasets. As a first step in generating the word clouds, we process textual annotations of each dataset to extract words and multi-word expressions. Out of all the words present in the dataset description we pick only nouns, adjectives and some other matching predefined patterns. Selected words are then normalized to ignore inflected forms (for example, gene, genes) using WordNet lemmatiser [55]. Besides single words, we also extract noun and adjective phrases. Syntactic analysis is performed using MedPost part-of-speech tagger [56].

Next, for a given group of datasets, we figure out a set of descriptive terms (words and phrases) that are over-represented in this group, compared to all the available datasets. We use hypergeometric *P*-value to identify such group-specific terms. The word cloud is then composed out of the terms with the lowest P-value. Within the word cloud, font size depicts their extent of over-representation of the term in the corresponding group of datasets.

## Abbreviations

ES: embryonic stem; GEO: gene expression omnibus; GO: gene ontology; KEGG: Kyoto Encyclopedia of Genes and Genomes; MCL: Markov cluster; MCM: mini chromosome maintenance; MEM: multi experiment matrix; MIAME: minimum information about a microarray experiment; ORC: origin recognition complex; preRC: pre-replicative complex; RMA: robust multi-array average.

## Authors' contributions

PA and MK implemented the resource. RK and PA developed the methods for the query. AT provided the annotation word clouds. PA, RK and JR performed the case studies. RK and JR drafted the manuscript. JV and HP conceived the study and provided general guidance. All authors read and approved the final manuscript.

## Additional files

The following additional data are available with the online version of this paper. Additional file 1 is a table listing datasets used for network reconstruction. The datasets were all on mouse platform Affymetrix U74Av2. In addition the analysis included an unpublished dataset that cannot be found in databases. Additional file 2 is a table listing datasets used for MCM complex study. Additional file 3 is a table listing the 116 genes that occur in more than two of the six cohorts of subunits MCM1-MCM6, where each cohort contains 100 probesets with most correlation relative to the corresponding subunit. Additional file 4 is a figure describing the selection of standard deviation cutoff. The figure shows correlation between number of significant query results and the number of datasets where the query gene standard deviation exceeds certain threshold. The maximal correlation is achieved when the threshold is 0.29.

## Supplementary Material

Additional file 1 The datasets were all on mouse platform Affymetrix U74Av2. In addition the analysis included an unpublished dataset that cannot be found in databases. Click here for file

Additional file 2 A table listing datasets used for MCM complex study. Click here for file

Additional file 3 A table listing the 116 genes that occur in more than two of the six cohorts of subunits MCM1-MCM6, where each cohort contains 100 probesets with most correlation relative to the corresponding subunit. Click here for file

Additional file 4 The figure shows correlation between number of significant query results and the number of datasets where the query gene standard deviation exceeds certain threshold. The maximal correlation is achieved when the threshold is 0.29. Click here for file
