# Supplementary figures and images for: Mining for coexpression across hundreds of datasets using novel rank aggregation and visualization methods
Source: Genome Biol. 2009 Dec 4;10(12):R139. doi: 10.1186/gb-2009-10-12-r139 (PMC2812946; doi:10.1186/gb-2009-10-12-r139)

## Finding the standard deviation threshold

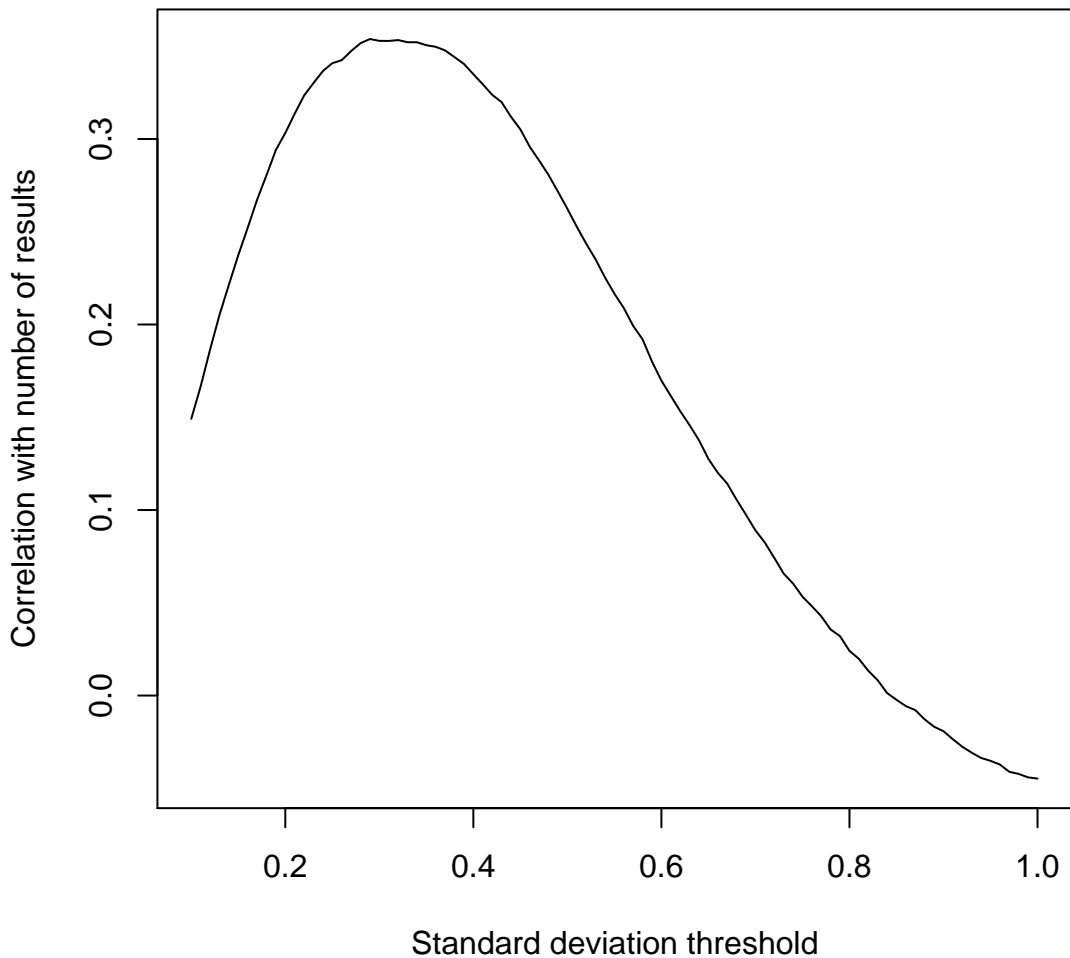

Supplement: Additional file 4 — The figure shows correlation between number of significant query results and the number of datasets where the query gene standard deviation exceeds certain threshold. The maximal correlation is achieved when the threshold is 0.29. [file gb-2009-10-12-r139-S4.pdf]
